# A Comprehensive Review of the Role of Virulence Factors in Enteropathogenic Escherichia coli-Induced Intestinal Injury

**DOI:** 10.7759/cureus.83475

**Published:** 2025-05-04

**Authors:** Owais M Ahmad, Seeme Rukh, Samuel Dos Santos Pereira, Ankita Saran, Vijay I Chandran, Alina Muneeb, Waldyr M Banderas Echeverry, Muyiwa Shoyoye, Damilare M Akintunde, Damilola Hassan, Zoya Morani, Lalain Masood

**Affiliations:** 1 Internal Medicine, Charles University, Hradec Kralove , CZE; 2 Internal Medicine, Malik Haider Hospital, Gujrat, PAK; 3 Internal Medicine, University of Buenos Aires, Buenos Aires, ARG; 4 Medical Microbiology, Postgraduate Institute of Medical Education and Research, Chandigarh, IND; 5 Internal Medicine, St. George's University School of Medicine, St. George's, GRD; 6 Medicine, Chaudhry Rehmat Ali Memorial Trust Hospital, Lahore, PAK; 7 Internal Medicine, Universidad Libre Cali, Cali, COL; 8 General Practice, Northern Ireland Medical and Dental Training Agency (NIMDTA), Belfast, IRL; 9 Medicine, Medecins Sans Frontieres, Magburaka, SLE; 10 Community Medicine/Public Health, Ahmadu Bello University, Zaria, NGA; 11 Nephrology, Newcastle upon Tyne Hospitals NHS Foundation Trust, Newcastle, GBR; 12 Family Medicine, Washington University of Health and Science, San Pedro, BLZ; 13 Dermatology, Bahria University Medical and Dental College, Karachi, PAK

**Keywords:** a/e lesion, intestinal injury, locus of enterocyte effacement (lee), type iii secretion system (t3ss), virulence factor

## Abstract

*Escherichia coli (E. coli) *is a rod-shaped gram-negative bacterium that includes the diarrheagenic strains, an identical group of intestinal pathogens.*E. coli *diarrhea is transmitted through the feco-oral route, through contaminated food and water.* Enteropathogenic E. coli *(EPEC)* *is one of the leading causes of diarrhea in the pediatric age group in developing and developed countries. Depending on the absence or presence of *E. coli* adherence factor plasmids, they are classified as typical or atypical isolates. The distinguishing feature of EPEC's pathology is the attaching and effacing lesions, which facilitate localized damage by tightly adhering to intestinal epithelial cells, disarranging their surfaces, and effacing microvilli. Typical EPEC possess the locus of enterocyte effacement (LEE), a pathogenicity island, encoding adherence factors, including the Type III Secretion System (T3SS), a needle-like structure injecting effector proteins into host cells. EPEC also have other effector genes like *cif* or *nleC* encoded by non-LEE pathogenicity islands, which enable destruction of tight junctions in the host cell. Another key virulence factor is bundle-forming pili (BFP), which aids in the first attachment to enterocytes. Methods like quantitative PCR exist to diagnose EPEC accurately. As of today, no licensed vaccine exists to prevent EPEC infections. Virulence factors for attachment, such as bfpA and intimin, and immunogenic carriers can be potential candidates for vaccine development. Moreover, studies are required to better understand the interaction of EPECwith the intestinal microbiome and immune evasion strategies. This article is aimed at providing a comprehensive review of the epidemiology, transmission, virulence factors, challenges in studying EPEC virulence factors, pathogenesis, host-pathogen interaction, mechanism of intestinal injury, diagnosis, treatment, antibiotic resistance, and vaccination strategy for EPEC, and future research implications. We conducted a comprehensive literature search using credible sources such as PubMed, Google Scholar, and Scopus. We refined our keywords, applied database filters, and assessed citations in the included studies. No meta-analysis, statistical aggregation, or formal evaluation of risk bias was carried out as this review consolidates the literature narratively. High-quality English articles published in reputable peer-reviewed journals from 2010 to 2025 were analyzed, and their findings have been summarized in this comprehensive review.

## Introduction and background

This article aims to review available information on the biology of enteropathogenic *Escherichia coli* (EPEC), the virulence factors, the pathogenesis and host-organism interaction, clinical symptomatology, detection and diagnosis, treatment implications, antibiotic resistance, and vaccination strategy. This article also aims to highlight future research directions on this topic. To achieve this, we used intestinal injury, type III secretion system (T3SS), locus of enterocyte effacement (LEE), attaching and effacing (A/E) lesions, and virulence factor as our search terms. Articles including reviews, clinical trials, and in vitro studies focusing on EPEC and the role of its virulence factors in intestinal injury published from 2010 to 2025 were reviewed, and findings were summarized and presented. 

*Escherichia coli* (*E. coli*) is a gram-negative bacillus. Hence, it stains pink under gram stains in light microscopy. *E. coli *is a rod-shaped (bacillus) bacterium. The organism is a fast lactose fermenter; hence, it would break down lactose into glucose and galactose, which will be further fermented to produce energy, often producing byproducts like lactic acid, carbon dioxide, or alcohol [1].

Other properties include* E. coli *Indole positivity, the ability of the bacterium to produce indole from tryptophan, an amino acid, through tryptophanase activity during metabolism. Virulence factors of *E. coli* include fimbriae, which can lead to urinary tract disease like cystitis and pyelonephritis, the P pili, and K capsule, which can lead to diseases such as pneumonia, meningitis, and LPS endotoxin, which can lead to septic shock. Additionally, *E. coli* is a facultative anaerobe. Thus, it can produce ATP via aerobic respiration if oxygen is present; however, it can switch to fermentation if oxygen is not present [2].

**Figure 1:**
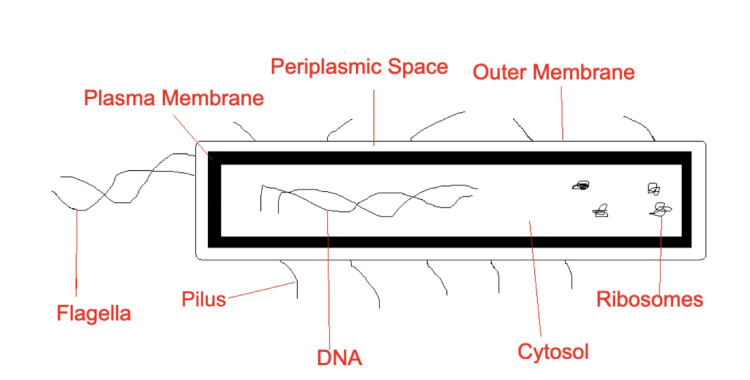
Diagram details a photo of E. coli and its structures Figure Credits: Owais M. Ahmad used jspaint.app to create this figure, which was accessed on 20 March 2025, inspired by reference [3].

The diarrhea-causing *E. coli *strains are an identical group of pathogens in the intestine, with various features including antigen structure, host colonization sites, the virulence mechanisms, and clinical evolution. Serological classification of the pathogenic *E. coli* is done using the antigens O (lipopolysaccharide) and H (flagella). Given the vast heterogeneity of these antigens (187 O antigens, 53 H antigens), serotyping is currently considered a laborious, costly, and unreliable diagnostic tool due to the cross-reactivity between different serogroups [4]. Most of the recent methodologies recommend the identification/classification of diarrhea-causing *E. coli* through molecular methods, which can identify the virulence factors of these strains [4]. Table 1 shows major diarrheagenic strains of *E. coli*.

**Table 1:** Details the various strains of E. coli Table Credits: Owais M. Ahmad created this table using Google Doc inspired by reference [5].

Strain	Toxin and Mechanism	Clinical Presentation	Type of Diarrhea	Produces Toxin
Enteropathogenic Escherichia coli	No production of a toxin. Adheres to the apical surface and flattens the villus. Prevents absorption.	Usually presents in children, hence leading to diarrhea in children.	Watery diarrhea (usually present in children)	No
Enteroinvasive Escherichia coli	Microbes invade the intestinal mucosa, which leads to further necrosis and inflammation.	Presents with invasive dysentery (this is an intestinal infection which leads to diarrhea containing blood or mucus)	Dysentery	Yes
Enterotoxigenic Escherichia coli	It produces two toxins, which are heat stable and heat labile enterotoxins. They cause no inflammation or invasion.	Watery diarrhea, which is commonly seen in travelers	Watery (common in travelers)	Yes
Enterohemorrhagic Escherichia coli O157:H7 is the most common subtype in the United States. O antigen refers to the lipopolysaccharide layer and H refers to the flagellin.	This bacterium is transmitted via undercooked meat, like ham burgers and raw leafy vegetables. Shiga toxin can lead to microthrombi formation, which leads to anemia, thrombocytopenia and decreased blood flow to the kidney; hence, causing renal failure.	This causes dysentery due to the toxin, which leads to necrosis and inflammation.	Dysentery	Yes

The feco-oral route is the main route of transmission whereby the pathogenic strains of the *E. coli* in fecal particles are passed from one person to another person's mouth [6]. Diagnosis of this bacterium includes utilizing a stool culture and subsequent antibiotic sensitivity testing. Treatment options include replacement of fluids and electrolytes. Antibiotic options are also available, including fluoroquinolones or rifaximin; however, rifaximin is ineffective against inflammatory enteropathogens. *E. coli* transmission prevention includes improving sanitation and hand washing recombinant cholera B subunit [7].

EPEC remain a major cause of infantile diarrhea in developing countries, with sporadic outbreaks of diarrhea due to it being reported recently in developed countries [8]. It was found to be the principal implicated organism and characterized for the first time, in the series of diarrheal disease outbreaks among infants in the United Kingdom between 1940 and 1950; the outbreaks were characterized by high mortality and morbidity among infants under six months of age [2]. Today, it remains of public health concern for children between 0 and 11 months in low- and middle-income countries, causing protracted diarrhea (moderate to severe), with mild fever and vomiting occasionally [9]. EPEC, after Rotavirus, may be the second most frequently seen etiology of diarrhea (25.4%) in the inpatient setting [10].

EPEC are defined by three key features: being able to cause diarrhea, the capacity to cause A/E lesions on the epithelium of the intestine, and the inability to produce Shiga toxins, heat-stable (ST) enterotoxins, and heat-labile (LT) enterotoxins. The bundle-forming pilus (BFP) and LEE are major virulence factors. BFP is an active fibrillar organelle that extends onto the bacteria surface and attaches to intestinal epithelial cells. Fourteen genes are required to form BFP in an 80 kb plasmid (pEAF), which generates the EPEC adherence factor (EAF). This causes adherence to the intestinal epithelial cells and a microcolony formation [11]. LEE encodes for a T3SS which is composed of three major components: (i) a needle complex in the outer membrane (EscC, EscD, EscF, EscL, and EscJ), (ii) an export apparatus present in the inner membrane (EscRST, EscU, and EscV), and (iii) a cytoplasmic sorting platform (EscA, EscK, EscL, EscN, and EscQ), which cumulatively lead to cytoskeletal rearrangements and increased permeability [12]. Other virulence factors include non-LEE effectors and additional virulence factors secreted via T3SS leading to disruption of tight junctions of the epithelium. LifA (lymphostatin) inhibits lymphocyte proliferation causing immunosuppression and flagella (H Antigen), which helps with motility and initial attachment [10,13]. Two types of EPEC have been described: the typical EPEC (tEPEC) and the atypical EPEC (aEPEC), based on the understanding of their virulence mechanism and genome [14,15].

Intestinal injury by EPEC is brought about by its attachment to the gut epithelial cells and the introduction of virulence factors that regulate the signaling pathways with consequent cellular disruption [16]. EPEC colonize the small intestine, specifically the duodenum, terminal ileum and Peyer's patches, targeting the complex network of tight junctions of the host cells, which serves as a critical barrier that regulates water and solute movement across the intercellular spaces [12,16]. The disruption of this complex leads to the compromise of the integrity of the intestinal epithelial layer, leading to the characteristic AE lesions and ultimately, to diarrhea and other symptoms.

## Review

Typical and atypical EPEC

Among the diarrhea-causing *E. coli* pathotypes, EPEC, ETEC, and EAEC are the most significant pathogens infecting children and causing diarrhea globally. EPEC are categorized into typical and atypical strains based on the presence of the eae gene within the LEE and the bfpA gene on the 'EPEC adherence factor' (EAF) plasmid. The EAF plasmid carries the BFP operon, crucial in the tEPEC pathogenicity. tEPEC strains possess eae+ and bfpA+, whereas atypical EPEC (aEPEC) strains possess eae but lack bfpA, i.e., eae + and bfpA- [17]. Some of the aEPEC strains might possess other virulence factors such as hemolysin, enteroaggregative heat-stable toxin (EAST1) not encoded on LEE [4]. The tEPEC is a leading etiology of diarrhea in infants in developing countries, whereas the aEPEC is an important etiology of diarrhea in developed countries [17].

**Table 2:** Comparison of different aspects of typical and atypical E. coli Table Credits: Alina Muneeb created this table using Google Doc Format inspired by references [14,17,18] *Brackets denote the frequent occurrence of nonmotile strains. **Recent changes in the trend of aEPEC-associated diarrhea cases are on the rise. LEE: Locus of enterocyte effacement; EAF: EPEC adherence factor

Features of *E. coli*	Typical *E. coli* (tEPEC)	Atypical *E. coli* (aEPEC)	
Frequently isolated EPEC strains	O55:[H6]*, O86:H34, O111:[H2], a O114:H2, O119:[H6], O127:H6, O142:H6, O142:H34	O26:[H11], O55:H34, O86:H8, O111ac:[H8], O111:[H9], O111:H25, O119:H2, O125ac:H6, O128:H2, O55:[H7],	
Virulence characteristics; production of virulence factors; adherence pattern	By LEE region and EAF plasmid; localized adherence (LA)	By LEE and other virulence factors such as enteroaggregative heat-stable toxin (EAST1), hemolysin, not encoded on LEE, localized-like adherence (LAL), diffuse adherence (DA), aggregative adherence (AA)	
Pathogenesis	More virulent	May be less virulent	
Diarrhea association	Strongly associated in children <1yr; adult infections are usually rare and associated with certain conditions	Less association previously**, usually in children.	
Reservoir	Only in humans	Human and some animals	

tEPEC shows stronger virulence and more consistent association with diarrhea due to both the eae gene and bundle-forming pilus (bfp) gene, enhancing its ability to attach to epithelial cells, causing diarrhea in 9/10 volunteers in challenge studies. aEPEC demonstrates reduced virulence compared to tEPEC because it lacks the bfp gene but contains the eae gene. It shows variable pathogenicity, with some strains causing diarrhea while others are found in asymptomatic individuals. Virulence may be enhanced in strains containing the efa1/lifA gene, which is associated with higher bacterial loads and stronger adherence to epithelial cells [19].

aEPEC is emerging as a colonizer of the intestine of children [18]. Furthermore, aEPEC has been found to possess an inherent ability to stay longer in enteric epithelial cells, disrupting normal cellular processes and potentially reducing apoptosis of intestinal epithelial cells. Some studies suggest that aEPEC may decrease intestinal apoptosis, possibly due to the absence of the bfpA gene, which may facilitate prolonged intestinal colonization compared to other intestinal pathogens. aEPEC is also significantly associated with endemic diarrhea, and outbreaks are also documented. How EPEC causes its invasive pathogenic effect is unknown, and this characteristic may contribute to the prolonged stay of these strains in the intestine leading to diarrhea under favorable host conditions [17].

Epidemiology of EPEC

Many factors contribute to the prevalence and variance of the epidemiology of EPEC such as age groups, geographic areas, and conditions based on socioeconomic status. Comparable studies have pinpointed that tEPEC is prevalent with infantile diarrhea, most notably in underdeveloped countries [4,20]. Over the years, tEPEC has been the catalyst for diarrhea in infant children within the first year of their lives. This association is higher within children six months and under. Countries such as Brazil, Chile, Mexico, and South Africa report that the number of infant diarrhea cases caused by tEPEC ranges from 30 to 40% [4]. Although tEPEC cases have declined in developing countries, instances of aEPEC infections have increased within these demographic areas. Utilizing Brazil as an example, 92% of EPEC isolates reported in 2001-2002 were of the atypical strain compared to 38% within the year spanning 1998-1999 [14].

According to a case-control study, which included 760 individuals, half of whom were positive for EPEC, EPEC-positive participants have a 4.03 times higher likelihood of developing gastrointestinal (GI) symptoms than the EPEC-negative population (P < .001) [21]. Between June and October, 70% of cases were reported, highlighting a correlation between seasonality and EPEC occurrences. According to the research in 2022, major risk factors for EPEC infections were international travel, accounting for an 11.8 times higher risk factor, and contact with symptomatic individuals, showing 16.5 times [21]. International destinations including Mexico and Central/South America were among the most significant risk factor locales, accounting for 45.7% and 21.7%, respectively [21].

Although a shift has occurred, tEPEC still stands as a notable pathogen in Africa and Asia, associated with mild to marked diarrhea in children under two years old [14]. In addition, various outbreaks of tEPEC infections have occurred in adults. Even though person-to-person contact remains the most prevalent avenue for transmission, the previously mentioned outbreaks were transmitted via contaminated food and water sources [14,20]. Contradictory to this, aEPEC infections occur in individuals regardless of age factor, including those adults currently diagnosed with HIV/AIDS [14]. Although worldwide occurrences have increasingly appeared, the specific part aEPEC has in diarrheal disease is still uncertain as it is often discovered in individuals experiencing both diarrheic and non-diarrheic symptoms [4,20]. Recent studies, in addition to outbreaks in Finland, the United States, Japan, and China, suggest that aEPEC potentially contributes to bloody and persistent diarrhea [14]. Challenges remain in diagnosing EPEC; however, molecular epidemiology has helped promote the distinction between tEPEC and aEPEC [14,20].

**Figure 2:**
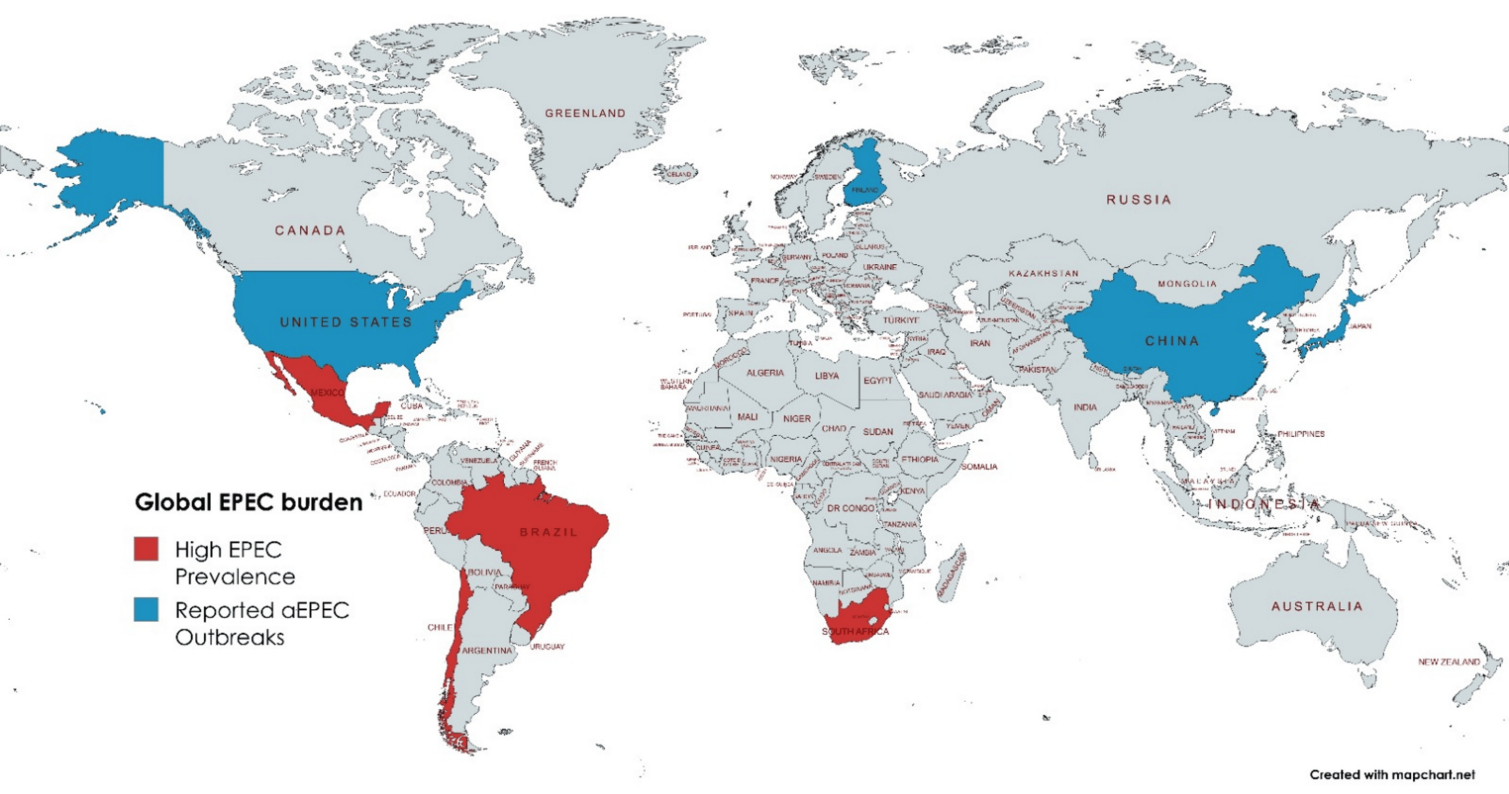
Global distribution of EPEC infections Figure Credits: Samuel Dos Santos Pereira created this map using the app MapChart, inspired by the references [4,14]. Based on epidemiological data, this map indicates regions with high EPEC prevalence (red) such as Brazil, Mexico, Chile, and South Africa. Recent aEPEC outbreaks (blue) have been identified in Finland, the U.S., Japan, and China [4,14]. aEPEC: Atypical enteropathogenic *Escherichia coli*

Barriers in outlining the global burden of EPEC

EPEC strains are widely distributed across developing countries and are recognized as a significant cause of diarrheal disease in infants and adults in these regions. Variations in geographic regions, periods, and socioeconomic status may also influence the epidemiology of EPEC-related diarrheal disease. Additionally, the failure to distinguish between tEPEC and aEPEC in certain studies further complicates such analyses [14]. In recent years, most diagnostic guidelines have moved toward using molecular techniques for classifying and identifying diarrheagenic *E. coli.* These methods are preferred as they can accurately detect specific virulence factors associated with these strains, revealing a higher burden of EPEC than previously reported. However, defining some pathotypes remains challenging due to imperfect gene targets, the emergence of mixed strains, and the isolation of these pathotypes from asymptomatic persons [22]. These complexities hinder accurate diagnosis and pose a significant epidemiological burden, as they complicate efforts to track and control the spread of pathogens.

In many developing countries, where EPEC infections were highly prevalent until the 1990s, some studies have not established a notable link between infantile diarrhea and tEPEC. For example, in Brazil, 92% of EPEC isolates from children between 2001 and 2002 were identified as aEPEC, a significant increase from 38% reported in a 1998 - 1999 study. However, contradictory data from other regions reports that tEPEC is the major cause of diarrhea compared to aEPEC [14]. Inconsistencies in studies like these further show the challenges in defining the global burden of EPEC, as variations in strain distribution, diagnostic methods, and study design contribute to inconsistent findings.

EPEC transmission

EPEC transmission is through the feco-oral route [23]. More specifically, EPEC may be transmitted directly through contact with human fecal matter or indirectly through contact with food, water, and fomites [24]. The two variants, tEPEC and aEPEC, are primarily similar in their transmission, as both forms spread between humans via the fecal-oral route directly and indirectly [14]. However, differences between tEPEC and aEPEC exist primarily regarding their natural reservoirs and preferred transmission modes. Humans are the only established reservoir for tEPEC, whereas humans and animals, such as cattle, dogs, and poultry, are reservoirs for aEPEC [14,25,26]. More specifically, tEPEC prefers direct modes of fecal-oral transmission, whereas aEPEC prefers indirect modes involving the environment to a greater degree.

The tEPEC variant preferentially spreads via direct contact between human carriers [14]. Transmission occurs in regions with poor sanitation and hygiene standards, where fecal matter is more likely to persist among people. Individuals are more likely to accidentally ingest the bacteria acquired from others in these areas, leading to potential tEPEC infection. While tEPEC can also spread indirectly via fomites, such as contaminated surfaces, most forms of indirect fecal-oral transmission, such as foodborne and waterborne transmission, are much rarer in tEPEC [14].

The aEPEC variant preferentially spreads to humans via indirect contact with food, water, and animals [14]. In regions with poor food-handling practices, aEPEC can spread via food by consuming raw or undercooked animal meat, pasteurized milk, and vegetables [14]. aEPEC can also spread via fecal-contaminated water sources [14]. Finally, livestock and wild animals play a significant role in transmitting aEPEC, as these animals affect the environment shared with humans and frequently contaminate food, water, and objects [14].

Virulence factors, environmental reservoirs, and survival strategies of *E. coli*

EPEC does not produce toxins. However, it depends on an advanced process known as "attaching and effacing" (A/E) to damage the epithelium of the intestine. An important virulence factor is BFP, which is encoded by the EAF plasmid. The BFP aids in the first attachment of enterocytes and the auto aggregation process. This enables the local adherence of EPEC to the enteric epithelial cells [27]. 

**Figure 3:**
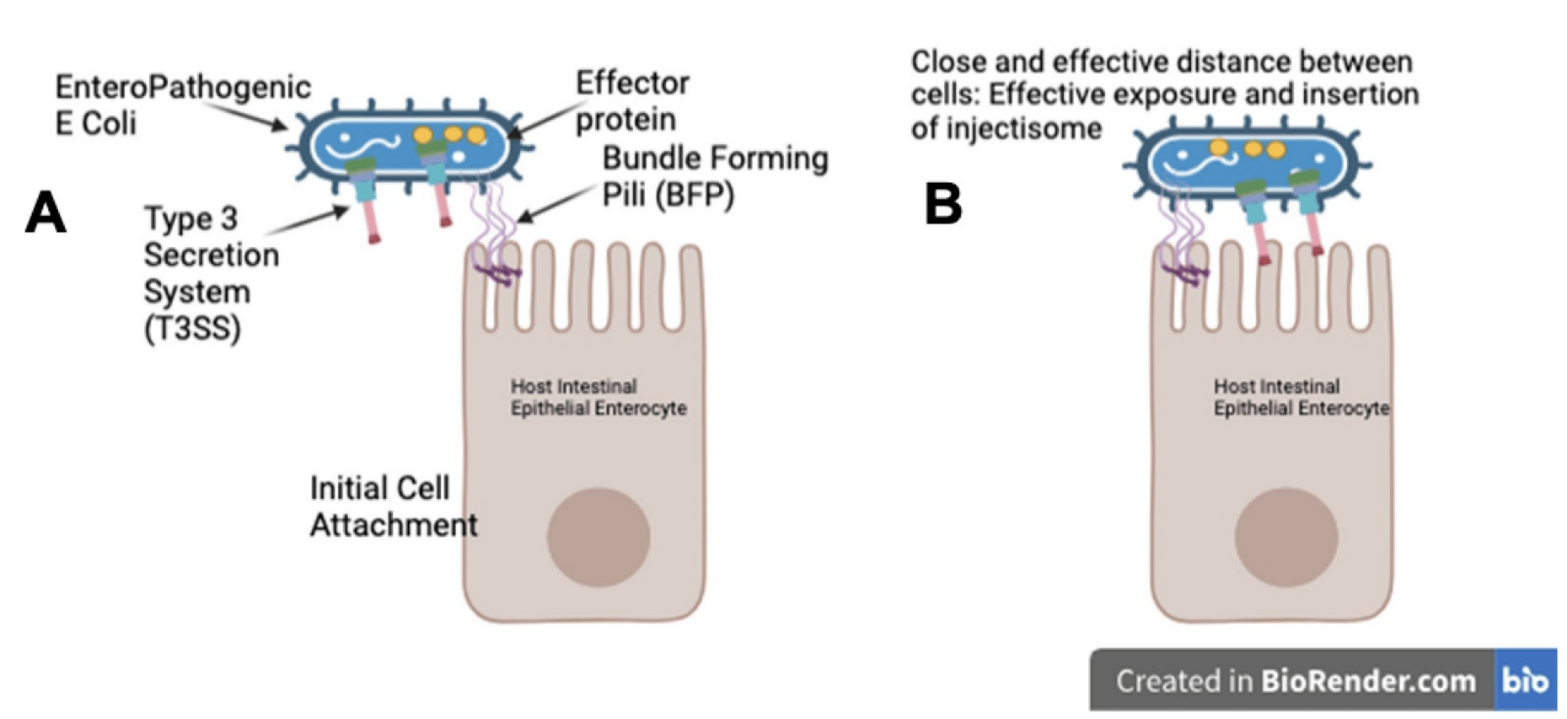
Figure showing how EPEC can use the BFP to allow for efficient effector translocation The figure shows induced pili retraction. A: Shows how EPEC can use the bundle-forming pili to allow for efficient effector translation; B: Shows close and effective distance between EPEC and the intestinal epithelial cells which allows for effective exposure. Figure Credits: Owais M. Ahmad created this figure using Biorender.com, accessed on February 11, 2025, inspired by reference [28].

Another virulence factor of EPEC and EHEC is Intimin, which is encoded by the eae gene. This functions as A/E proteins [29]. It works as an outer membrane adhesion protein, which binds to Tir (translocated intimin receptor). Tir and 25 other bacterial proteins are injected directly into the enterocyte of the intestine by a type III secretion. Once within the cytoplasm of the intestinal enterocyte of the host cell, this is inserted into the plasma membrane enabling surface exposure and intimin binding. Tir-intimin binding allows for tight bindings of EPEC and EHEC to intestinal enterocyte epithelium, leading to effacing lesions on intestinal enterocyte epithelium [30].

EPEC has a T3SS encoded by the LEE pathogenicity island, a distant class of genomic island acquired by EPEC by horizontal gene transfer. The T3SS acts as a molecular injector, allowing various T3SS bacterial effectors into the intestinal enterocyte epithelium/host cells. One of the effector proteins is EspF, exclusive to EPEC and EHEC and acts to disrupt the epithelial barrier, anti-phagocytosis, microvillus, and modulate the cytoskeleton [30]. 

**Figure 4:**
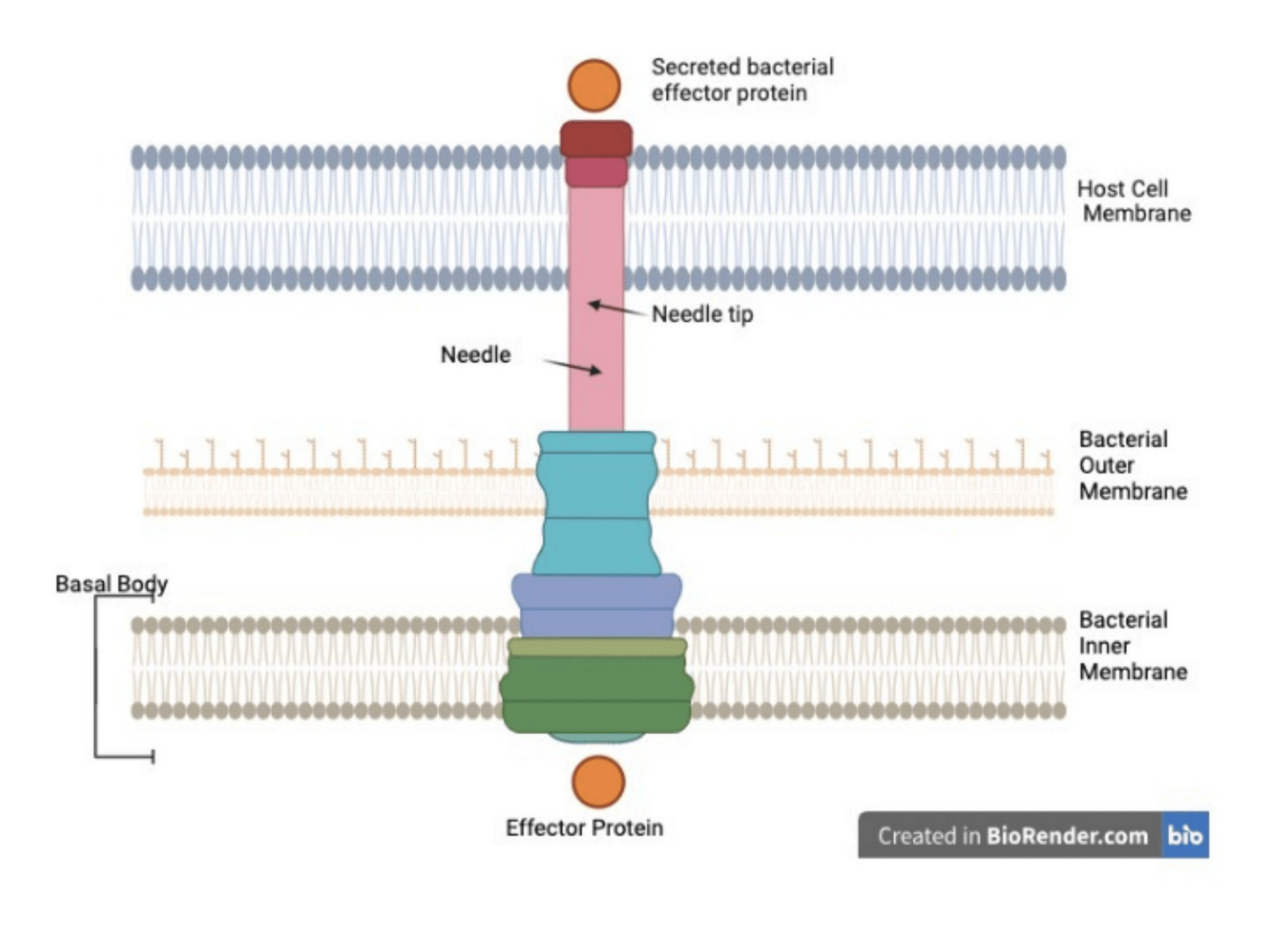
The type III secretion system Figure Credits: Owais M. Ahmad used Biorender.com to create this figure, which was accessed on 11 February 2025, inspired by Reference [31].

The effector proteins called mitochondrial associated proteins (MAPs) are then imported into the mitochondrial matrix, leading to increased mitochondrial permeability, hence inducing apoptosis. EsoG and EsoH lead to disruption of microtubules, hence, damaging the cytoskeletal structure [32]. Apart from the LEE region, EPEC also has effector genes such as cif, esp/nleA, NLRB, nleC, nleD, nleE, and nleF that are arranged in six pathogenicity islands and are coded by non-LEE (nLE). These cause immune evasion by causing changes in the cytoskeleton and interrupting the inflammation pathways of the host's body by inhibiting pro-inflammatory transcription factor NF-KB, which helps to evade immune response and allow persistent colonization [4,33]. Besides, NleA, EspF, and Map, non-LEE-coded proteins also destroy tight junctions within the host intestine [34].

**Figure 5:**
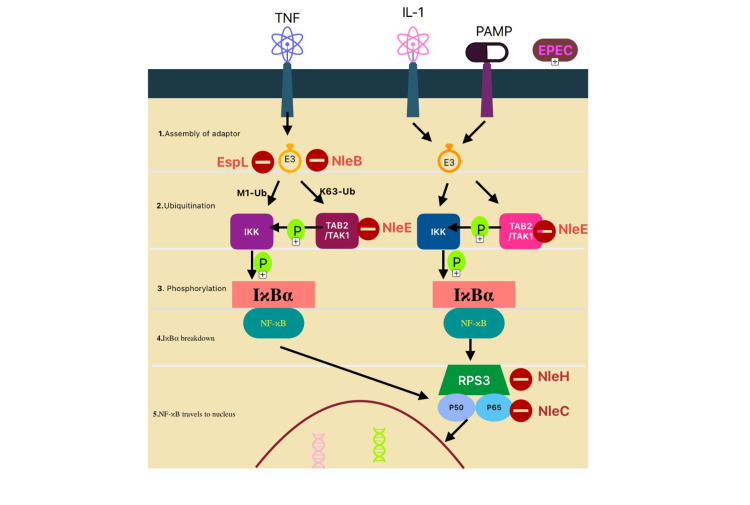
Stepwise presentation of the NF-kB signaling pathway Figure Credits: Seeme Rukh made this figure using Free Form Apple Macbook Application inspired by reference [35]. This signaling pathway is activated during enteropathogenic *Escherichia coli* (EPEC) infections. Events include EPEC-mediated activation triggered by pathogen-associated molecular patterns (PAMPs) and interleukins. The process starts with the E3 ligand followed by the role of M1 and ubiquitin chains attached to K63 and then phosphorylation by IKK and TAK, breakdown of IκBα, and then NF-Κb, which is a transcription factor, followed by entry into the nucleus. Effector proteins in E. coli such as EspL, NleB, NleC, NleE, and NleH1/2 interfere with the steps shown in the figure [35].

Bacteria must quickly detect and make alterations to changes in pH, oxygen, and nutrient availability. These changes need energy and are controlled by transcription [36]. *E. coli's* ability to maintain iron balance through heme receptor allows it to thrive in the intestine more effectively than other organisms [30]. *E. coli *in anaerobic conditions assume a filamentous appearance (nearly 70%). This is due to a fall in RNase E protein in low oxygen states. This is related to enolase-bound RNA degradosome activity which supports DicF. So, some strains of *E. coli* change to the form of a filament from the rod under stress conditions [37]. DicF activates pchA translation under low oxygen conditions, such as in the gastrointestinal tract, causing increased LEE expression [38].

**Figure 6:**
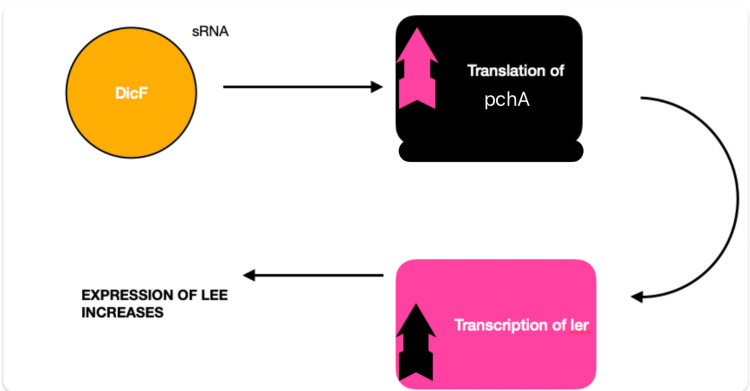
Changes that occur in E. coli in low oxygen states Figure Credits: Seeme Rukh made this figure using Pages Apple Macbook Application inspired by reference [38].

To avoid being trapped by immune defenses, bacteria must express flagellar genes which help in motility. In EPEC types, the predominant antigens of flagella are H2 and H6, additionally there are also known H types (mainly H7, H8, H9, H12, H21, H25, H27, and H34) that are rare, and a few strains of EPEC are termed as being H negative, i.e., they are not motile [4].

Other virulence factors of other serotypes of *E. coli* include fimbriae, which can lead to cystitis. Fimbriae are hair-like and thin projections extending from the bacterial surface. They are composed of protein and function to adhere to surfaces including host tissues. This plays a key role in adhesion colonization and infection and biofilm formation. Additionally, P-pili can lead to pyelonephritis. Moreover, pili, otherwise known as "sex pili" or "conjugative pili", these structures are like fimbria, but they are longer and thicker. Pili are involved in the conjugation; hence, the horizontal gene transfer in bacteria promotes the exchange of genetic material between bacteria. In addition, the K capsule is responsible for causing pneumonia, neonatal meningitis, and LPS endotoxin can lead to septic shock [2].

Challenges in evaluating the virulence factors

According to the information collected in the studies analyzed, it can be inferred that there are several virulence factors related to EPEC that make studying these very challenging. First, EPEC strains have non-conventional virulence factors, such as the EAF plasmid. For this reason, the identification and characterization of strains with potential damage are complicated [39]. Another problem is the difficulty of controlling the pathogenicity island where the LEE is located. It is known that the LEE has complex regulations and environmental influence, which in turn has a fundamental function in the coding of essential proteins. For this reason, it is difficult to identify the specific genes that are controlling accurately [2]. Furthermore, it has been found that there are shared functions in several effectors of the T3SS, which makes it difficult to precisely identify each protein during the infection process [2].

In addition, there are in vitro limitations when studying the mechanisms of virulence factors in cell cultures; it is considered that these are not exactly reproducible when compared to what occurs in vivo systems due to the recognized complexity that this represents. For this reason, representations that are not completely accurate could be generated [40]. Finally, it has been determined that the number of animal models that have been used, generally in mice infected with Citrobacter rodentium, in an attempt to determine the pathogenesis of EPEC is not the most appropriate, since it may not accurately imitate the disease in humans due to the difference in the many virulence factors and the host's immune response that play a predominant role [39].

Pathogenesis

The widely known gut commensal flora *E. coli *can become pathogenic through genetic variation and attainment of virulence factors. After acquiring the pathogenicity island LEE, an espC gene that encodes a serine protease enzyme, *E. coli* evolved into EPEC [5]. Pathogenesis underlying EPEC-associated diarrhea and subsequent intestinal injury can be understood in the following steps: (i) Attachment to enterocytes: EPEC locally adheres to small intestine epithelial surface and results in microcolony formation. It is seen in typical EPEC strains, driven by EAF plasmid, which encodes for the BFP, a major virulence determinant for EPEC. This attachment occurs in the form of loose aggregates with atypical EPEC infection, with the help of effectors like EspA and the common pili of *E. coli* (as it lacks pEAF) [41]. (ii) Type III secretion system (TTSS) injects the host cells with effector molecules. The LEE pathogenicity island encodes for TTSS in EPEC & EHEC, which forms a channel for effectors like Tir and others to be delivered into host cells. (iii) Strong intimate attachment via Tir-intimin interaction: Tir undergoes modifications by tyrosine-protein kinase before being embedded into the host membrane. The bacterial intimin adhesion molecule forms a complex with Tir and other effectors like EspB, EspD and EspA. (iv) Actin depolarization, loss of microvilli, and pedestal formation: The distinctive histopathological signature in EPEC pathogenesis is the formation of A/E lesions crucial to the intestinal injury are caused by EPEC. Following the intimin interaction (attachment), effector proteins EspG, EspH, EspF, Tir, and MAP interact with Arp 2/3 & N-WASP host molecules. This results in rearrangement of the cell cytoskeleton, actin depolarization underneath the attached bacteria and resulting formation of a cytoplasmic protrusion called the pedestal. The subsequent microvilli loss (effacement) interferes with the absorptive area, causing inflammation, and water and electrolyte loss [5,10,42]. (v) Breaking intestinal epithelial tight junctions: multiple EPEC effectors contribute to breaking the epithelial barrier. EspF interacts with ZO-1/ZO-2, capturing profilin, and MAP modifying occludin & claudin junction proteins. The synthesis of new proteins from the ER to the Golgi is directly interrupted by effectors NleA & EspG through their interaction with Sec24 & GTPases Arf1/6 respectively. EspG1//G2 alters microtubules and disrupts epithelial junctions. They also prevent repairs by disrupting the calcium switch mechanism by interfering with calmodulin signaling pathways. EspF and EspG sequester aquaporin 1 and 2, disrupting water absorption. All these above mechanisms result in watery diarrhea and intestinal injury [33,43].

**Figure 7:**
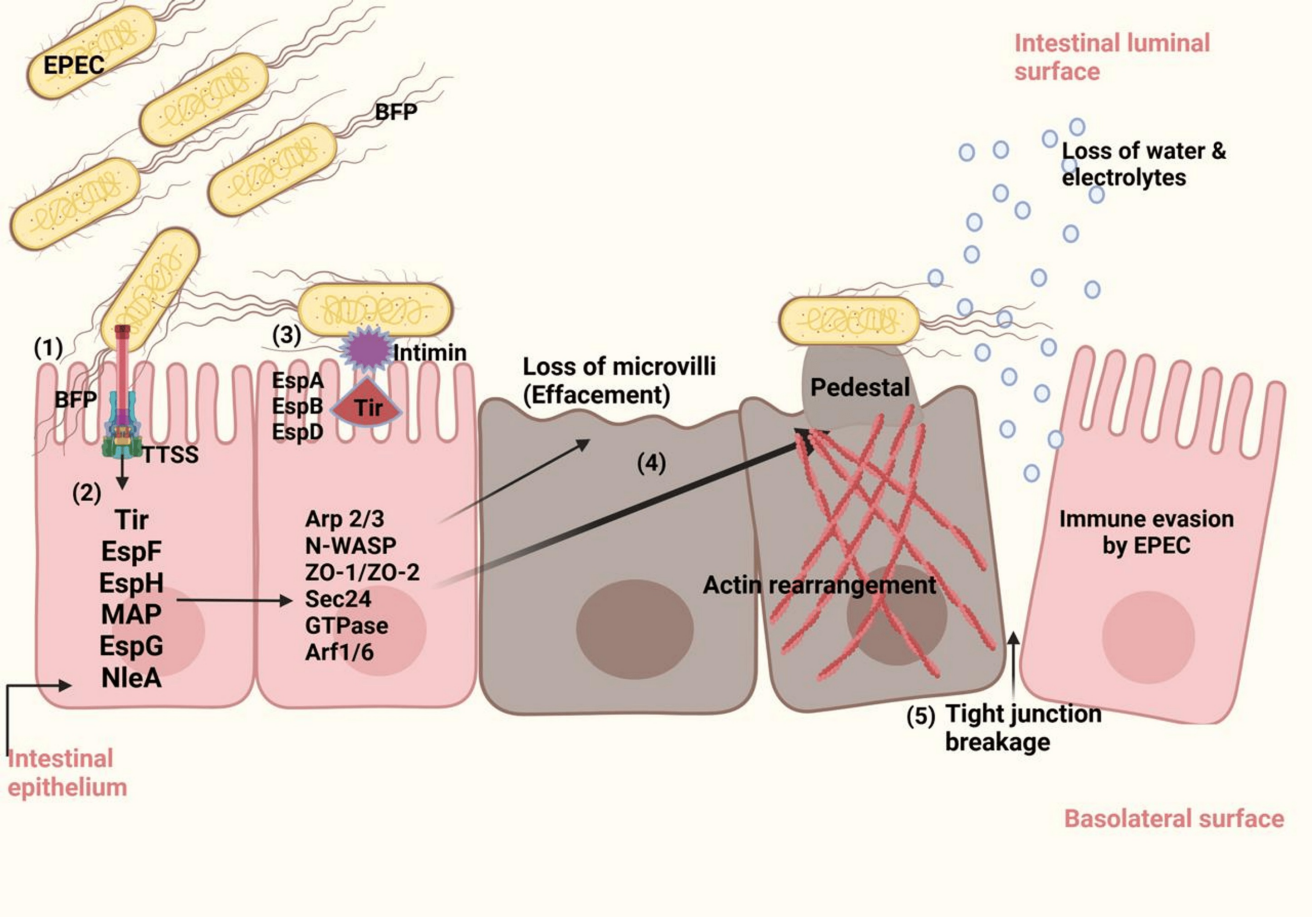
Pathogenesis of EPEC-causing diarrhea and intestinal injury Figure Credits: Ankita Saran created this figure using Biorender.com, which was accessed on February 9, 2025 inspired by references [5,10]. (1) EPEC attachment to epithelial cells of the intestine by BFP. (2) Effector molecules are injected into host cells via a type III secretion system (T3SS). (3) Strong intimate attachment via Tir-intimin interaction. (4) Actin depolarization, loss of microvilli, and pedestal formation. (5) Breaking intestinal epithelial tight junctions [5,10]. EPEC: Enteropathogenic *Escherichia coli*;* *BFP: bundle-forming pilus

Also, some researchers have argued that pedestal formation plays a key role in resisting phagocytosis, promoting colonization, and enhancing the motility of EPEC-forming macrocolonies [42]. EPEC evades immune response via effectors NleA, NleB, and NleF. This hinders NLRP3 assembly, suppresses caspase-4 & 8, and NF-κB signaling, dampening inflammasome activation, immune recognition, and effective inflammatory response [44].

Host-pathogen interaction

Intestinal host cells employ various defense mechanisms against microbial infections. Tight barrier junctions seal adjacent epithelial cells and serve two key functions: (i) selectively controlling ion and solute passage through the intercellular space and (ii) acting as a barrier to prevent the mixing of basolateral and apical membrane components, thereby keeping the cell polarity. Ion transport systems help to maintain proper ion balance and includes sodium-D-glucose cotransporter (SGLT-1), Na+/H+ exchanger (NHE3) and Cl-/HCO3- exchanger (DRA). Water regulation is through aquaporins-2 and -3, which control water absorption and also help prevent excessive fluid loss [43].

The gut mucosal immune system is stimulated occasionally by antigens from external pathogens, from the diet, and resident flora, which are periodically activated by antigens from external pathogens, food, and the resident flora in the colon and small intestine. The normal flora of the gut keeps a diverse population of well-balanced bacterial species, regulating local immune function through interactions with gut-associated lymphoid follicles, T-cells, and antimicrobial peptides. Antimicrobial peptides such as human beta defensins (hBD3) recruit monocytes in an isoform-dependent manner [45].

A balanced interplay between antimicrobial peptides and gut microbiota is observed in healthy individuals, where innate immune receptors in epithelial cells recognize peptidoglycan in gram-negative bacteria to maintain intestinal homeostasis. Toll-like receptors (TLRs) recognize pathogenic and commensal bacterial ligands, activating immune responses that protect the intestine from injury and facilitate host-microbial interactions. The interplay between gut flora and TLRs regulates local inflammation and maintains intestinal homeostasis. Therefore, the absence of intestinal microflora leads to insufficient antibody response and a compromised immune response to the pathotypes and intestinal inflammation [45].

In addition to the aforementioned immune response, the gut mucosal immune system comprises the secretory immunoglobulin A (IgA) that constitutes a local and systemic immune response. IgA primarily neutralizes the toxins and gut pathogens, thereby maintaining the intestinal host-microbial homeostasis [45].

EPEC can overcome these defenses through its various effector proteins, leading to infection and diarrhea. EPEC causes disequilibrium in the host cells' electrolyte transport, and the dysregulation of these transport pathways, is responsible for the rapid onset of the symptoms. The effector proteins of EPEC, namely, NleA and Map, interact with Na+/H+ exchanger regulatory factor 2 (NHERF2) and alter its function, leading to decreased Na+ uptake. In intestinal epithelial cells, the expression of NHE2 and NHE3 is restricted to the apical surface, whereas NHE1 is expressed on the basolateral membranes. EPEC infection in in-vitro models was shown to activate NHE2 but inhibited the NHE3, the key Na+ absorbing transporter. EPEC has also been shown to inhibit the function of the sodium-D-glucose transporter (SGLT1), a major contributor to fluid uptake in the small intestine and hence could contribute to diarrhea [10]. Moreover, by modulating the AQP (epithelial aquaporin) expression, the water transport is directly altered by EPEC [4].

EPEC-induced diarrhea is characterized by compromised host cell tight junctions, and research implicates effector proteins EspF, Map, EspG1/G2, and NleA in this disruption. Specifically, EspF's N-terminal region targets mitochondria and nucleoli, altering their function, while its C-terminal region interacts with SNX9 and NWASP, regulating actin polymerization via the Arp2/3 complex. EspF may recruit zonula occludens-1 and -2 into actin pedestals and disrupt tight junctions by internalizing claudin-1, -3, and -5. Another EPEC effector protein, Map, interacts with EspF and disrupts tight junctions. Map modulates mitochondrial processes and acts as a guanine-nucleotide exchange factor for Cdc42 GTPase, promoting filopodia formation. Map's C-terminus interacts with the Na+/H+ exchanger regulatory factor 1, linking with ezrin and promoting interaction with the actin cytoskeleton. The effector protein NleA disrupts tight junction proteins zonula occludens-1 and occludin, increasing paracellular permeability. The tight junction proteins, the zonula occludens-1 and occludin, are disrupted by NleA leading to increased paracellular permeability [10].

The inflammatory response is believed to play a more significant role in the severity and duration of the disease rather than its initial stages. Elevated levels of IL-1β, TNFα, and interferon (IFN) γ in the infected mucosa are characteristic of EPEC infection. Both pro-inflammatory and anti-inflammatory pathways in epithelial cells contribute to the inflammatory response mediated by EPEC. The EPEC secreted components are responsible for the pro-inflammatory response, while the effectors inserted in the host cells by the T3SS attenuate the inflammatory response [4].

In the small intestine, EPEC can adhere to both absorptive cells and microfold cells (M cells) within Peyer's patches. Given the proximity of Peyer's patches to host immune cells, including B cells, T cells, dendritic cells, and macrophages, EPEC triggers acute immune responses as early as 12 hours post-infection. M cells facilitate EPEC uptake via endocytosis, delivering them to resident macrophages in the subepithelial space. However, EPEC employs various strategies to evade phagocytosis, including secreting T3SS effector proteins. For instance, EspB disrupts pseudopod extension and phagosome closure in macrophages, while EspF inhibits phosphoinositide 3-kinase (PI3K)-dependent F-actin rearrangement. Additionally, EspJ prevents opsonization with immunoglobulin G (IgG) and complement component iC3b. EspH can repress the activation of Rho guanine nucleotide exchange factors (RhoGEF), which regulates actin rearrangement [8].

A previous study showed that EPEC can produce "CORE", a LEE-encoded T3SS injectisome component, to obtain nourishment from host cells. CORE can facilitate the production of another membranous nanotube directly extracting nutrients from the host cell cytoplasm. Studies have demonstrated that EPEC clinical isolates can acquire nutrients via a CORE-dependent mechanism, whereas the non-pathogenic *E. coli *(K12 strain) lacks this ability. Different environmental factors such as nutrient limitations, immune response, pH, and so on can stimulate stress responses in bacteria, causing virulence factors expression at the appropriate time and place. For example, the stomach's extremely acidic environment (pH 1.5-3.0) activates bacteria's acid resistance system, enabling survival for approximately two hours. Pathogenic *E. coli, *including EPEC, possess four acid resistance systems (AR1-AR4) to counter acidic environments. Additionally, under nutrient-limited conditions, bacteria exhibit a "stringent response," facilitated by guanosine tetraphosphate (ppGpp). This response can also be triggered by other stressful conditions in the host gut environment, and its role in pathogenesis has been established in various bacterial species. However, only a few studies have reported the function of stringent response in both EPEC and its host immune response [8].

EPEC-induced intestinal inflammation and injury

The mentioned virulence factors lead to intestinal changes involving the effacement and destruction of microvilli, further leading to a decrease in the absorptive surface area. Additionally, tight junction disruption causes increased permeability and fluid loss. Once effector proteins enter the intestinal epithelium enterocytes, they cause rearrangement of the actin filaments, which leads to the formation of the pedestal. This rearrangement leads to a pedestal formation beneath the bacterial cells, which disrupts the intestinal barrier's normal function and exacerbates diarrhea [46].

Furthermore, EspF and EspG effector proteins disrupt tight junctions causing intestinal permeability and the Zonula occludens toxin (ZOT) leads to leaking ions and water further worsening the diarrhea. Moreover, damage to the epithelial cells decreases the amount of glucose and sodium absorption, further increasing the fluid loss [47]. EPEC causes host inflammation by activating NF-KB, which increases pro-inflammatory cytokines such as IL1B, IL-8, and TNFA. IL-1 leads to the recruitment of leukocytes, specifically more neutrophils at the site of inflammation. Additionally, there is more formation of ROS that also damages epithelial cells [32].

Clinical manifestations

Infections stemming from EPEC can appear as sudden or continuous diarrhea, mainly in children who are less than two years old. In general, acute watery diarrhea is the common presentation of EPEC and frequently coexists with pyrexia, emesis, and hypovolemia. Symptom onset is swift and often appears acutely following consumption. Chronic diarrhea may lead to hypovolemia and deficiency of micronutrients [4]. Clearly defining the clinical characteristics of EPEC infections is arduous as some studies have shown that it is commonly detected in mixed intestinal infections [4]. Moreover, EPEC carriers who are asymptomatic have been found, particularly among aEPEC strains [20]. Studies from the Netherlands, Peru, and Germany have discovered EPEC in gastroenteritis patients and asymptomatic persons alike, with certain studies signaling similar frequency in these two groups of individuals [20].

Host susceptibility subject to genetic factors, immune reactions, and the role of the gut microbiome is one of several hypotheses that aim to explain why EPEC is detectable in asymptomatic carriers [20]. In addition to this hypothesis, bacterial variation, defined by the fact that various EPEC types have distinct virulence genes, may help us determine if the infection will cause disease symptoms [20].

Diarrhea is the predominant manifestation in symptomatic infections, accounting for roughly 97% of cases. Other manifestations often reported are emesis, pain in the abdomen, and pyrexia at 34%, 53%, and 18%, respectively. Frequently, patients refer to abdominal pain characterized by cramping or sharpness, with children 18 and under being febrile. The severity of diarrheal disease varies with the average number of episodes between 4 and 5 and some cases report up to 40 episodes of defecation in 24 hours. In 83.8% of related cases, acute diarrhea occurs while persistent diarrhea is experienced at 16%, lasting 7 to 18 days based on the patient's age [21].

The severity of diarrhea in symptomatic individuals indicates a higher bacterial load, which correlates with EPEC in the stool sample, as opposed to asymptomatic individuals. Within this construct, age plays a role as older adults commonly have fewer severity cases. Amongst symptomatic patients with persistent diarrhea, 18% of them require follow-up visits while 23% of these cases require hospitalization [21].

Comparison with other enteric pathogens

In many studies, EPEC has been compared to other enteric pathogens in terms of the mechanism of causing infection and interaction with inflammasome pathways. Gastric epithelial cells are damaged by Pseudomonas, *Helicobacter. pylori*, and EPEC, which cause hospital-acquired infections, gastritis, and diarrhea, respectively [32].

**Figure 8:**
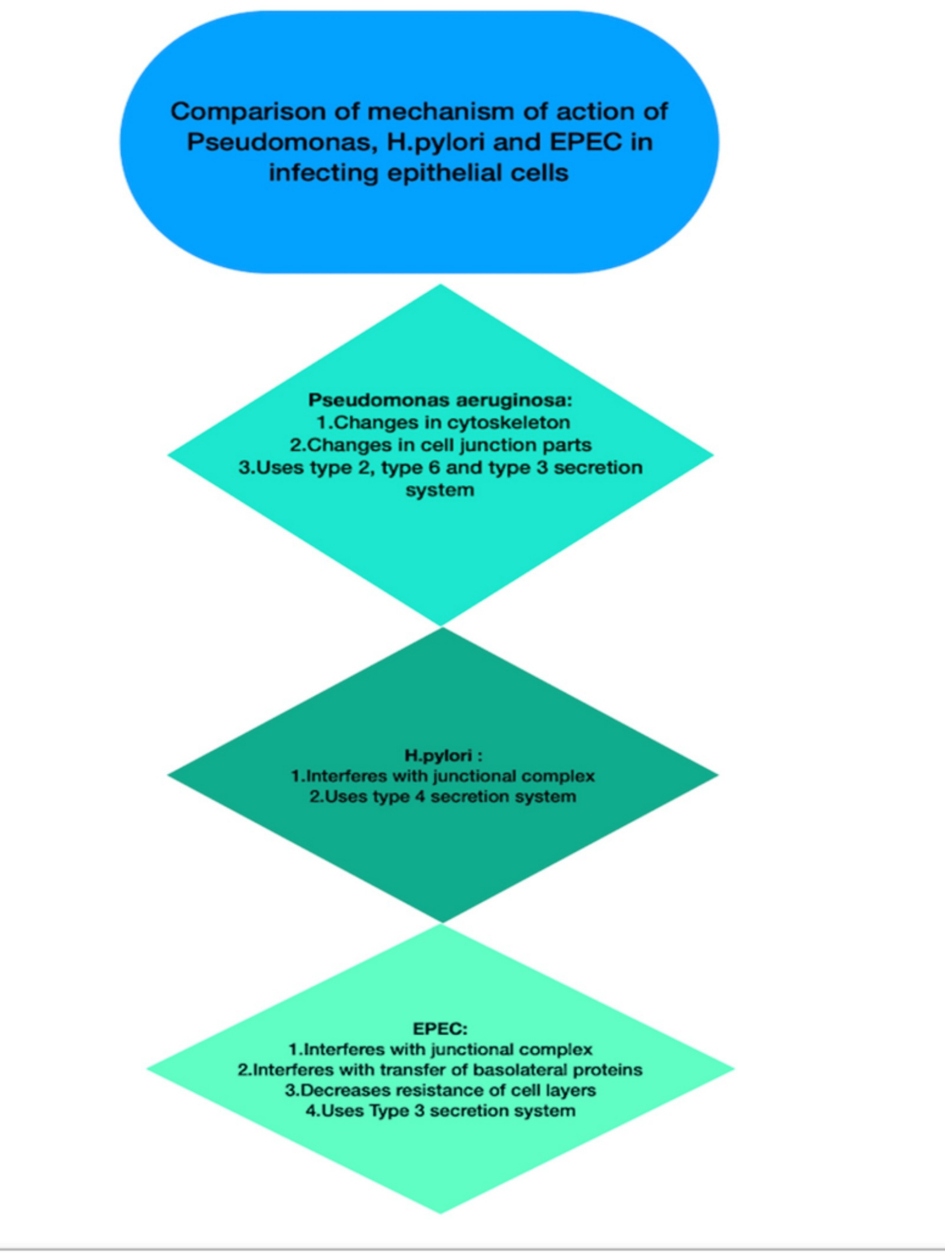
Comparison of the mechanism of action of enteric pathogens Figure Credit: Seeme Rukh made this figure using Pages Apple Macbook Application inspired by reference [32].

Enteric bacteria interact with inflammasome pathways, which activate the process of apoptosis, which is programmed cell death (mainly of cells containing bacteria). Some enteric pathogens such as Listeria monocytogenes and H. pylori benefit from inflammasome pathways while others such as E. coli, Salmonella, Shigella, and Yersinia interfere with inflammasome pathways using the T3SS [48]. Salmonella enterica has a T3SS as in EPEC, SPI-1 is the pathogenicity island related to infection by Salmonella in the small intestine [49]. In Salmonella typhimurium, SlrP prevents initiation of inflammasome pathway [50]. Meanwhile, Nle in EPEC attaches to Caspase-4 and inhibits apoptosis [51]. Shigella, another enteric pathogen, avoids cell death by OspC3 which acts on Caspase 4 [52]. Pyrin inflammasome activation in cases of Yersinia infection is prevented through a virulence factor YopM [53]. Listeria is known to cause activation of NLRP3 inflammasome and AIM2 inflammasome via LLO (listeriolysin O) and by releasing DNA in cytosol, which causes a release of interleukin-18 and neutrophils, which attack the host's immune system and aid Listeria in thriving [54]. H. pylori have a type 4 secretion system while EPEC has a type 3 one. NLRC4 inflammasome decreases the production of antimicrobial peptides and helps H. pylori to survive [55].

Detecting and diagnosing EPEC

In the modern day and age, it is impractical and uncalled for to investigate and diagnose all cases of diarrhea, since the majority resolve spontaneously. However, EPEC causes pediatric watery diarrhea, which is often persistent, and is usually investigated in traditional clinical microbiology laboratories. The stool samples for patients are cultured on classical culture media like MacConkey agar (MA). The isolated flat lactose-fermenting colonies of *E. coli* on agar media can be subjected to agglutination reactions on glass slides using antisera against classical serogroups of EPEC. The main benefits of slide agglutination are methodical ease and availability in laboratories globally. On the other hand, drawbacks include difficulty differentiating between tEPEC and aEPEC, and cross-reactivity, stemming from the high variability of serogroups. Additionally, falsely identifying as negative is common as certain EPEC strains fall outside the traditional serogroup classifications [4,14].

Various immunological tests for detecting EPEC from clinical samples have been defined in published literature. These tests usually target the specific virulence proteins of EPEC like BFP, intimin, and secreted proteins like EspB among others. Immunoblotting and immunofluorescence tests based on monoclonal/polyclonal antibodies targeting BFP have been developed with a sensitivity of 83 to 92% and specificity of 96 to 97% [14,56]. An immunoblot assay utilizing polyclonal antibodies produced in rabbits against intimin protein has demonstrated 97% sensitivity and near-perfect specificity in detecting tEPEC strains [14]. Rapid tests like immunochromatographic or card tests, which can be used in rural and poor resource settings, have proven to help detect EPEC by targeting EspB. Other methods based on EspB are latex beads tagged with monoclonal antibodies against EspB, which causes agglutination. Serological methods are preferred in general during outbreak investigations and epidemiological studies [4,14].

**Table 3:** PCR targeting genes for detection of EPEC Table Credits: Ankita Saran made this table on an MS Word Document inspired by reference [4]. PCR: Polymerase chain reaction; tEPEC: typical EPEC; aEPEC: atypical EPEC; eae: E. coli attaching and effacing gene; bfp: bundle-forming pilus gene; stx: Shiga toxin gene

PCR gene targets	tEPEC	aEPEC
Eae	+	+
Bfp	+	-
Stx	-	-

For more accurate diagnosis of EPEC, molecular methods like the polymerase chain reaction (PCR) should be used. Both conventional and real-time quantitative PCR (qPCR) have been standardized and validated in published literature. Once eae gene positive and stx gene negative isolates are identified, they are subsequently subjected to bfp gene targeting PCR to help differentiate tEPEC from aEPEC [22,57].

The recent advances in PCR technology have resulted in many commercial multiplex PCR panels being developed like Biofire and Luminex. These panels have a syndromic approach in identifying common culprits (bacteria, viruses and parasites) of gastroenteritis like EPEC, EHEC, ETEC, EAEC, Clostridioides difficle, Cryptosporidium, Shigella, and Salmonella among others. While panel testing offers the advantage of covering a variety of etiology, high sensitivity, and automation, the results must be interpreted with caution and must be correlated with clinical symptoms, as carrier states are seen among endemic areas and can pick up very low concentrations of bacteria. Other newer diagnostic tools include digital droplet PCR and Taqman Array Card test, which are rapid automated techniques offering more advantages [22,57].

Treatment, antibiotic resistance, and vaccination strategies for EPEC

The primary treatment for diarrheal diseases is fluid replacement, either oral (water and electrolytes) or intravenous, with probiotics or antimotility agents if needed. In most cases, including EPEC-induced gastroenteritis, it is self-limiting and resolves without additional intervention. Therefore, antibiotics are not routinely prescribed for the treatment of watery diarrhea. However, persistent diarrhea lasting beyond 14 days, especially in children, and accompanying alarming symptoms like blood in stools, fever, immunosuppressed patients, and signs of severe dehydration warrant the use of antimicrobials. The rising antibiotic resistance among tEPEC and aEPEC strains has been reported globally, reasons for this could be the gain of resistance genes via horizontal gene transfer through plasmids and transposons, varying guidelines for prescription, control, and antimicrobial susceptibility testing across clinical laboratories worldwide [4,58].

Published literature depicts resistance in EPEC for most of the first-line antimicrobials like penicillin, cephalosporins, fluoroquinolones, trimethoprim-sulfamethoxazole, and aminoglycosides [4]. Atypical strains are relatively less resistant than tEPEC, although this trend is changing [59].

Studies have reported that the percentage of EPEC infections with resistance to three or more classes of antibiotics is also emerging. Global studies report ampicillin resistance ranging from 51% to 100%. Similarly, resistance rates vary between 11% and 66% for cefotaxime and cefuroxime, 0% to 45% for ciprofloxacin, and 25% to 83% for trimethoprim-sulfamethoxazole [4]. However, last-resort options like carbapenems, fosfomycin, and colistin remain effective for EPEC, retaining susceptibility [60].

As of today, no licensed vaccine exists to prevent EPEC infections. Virulence factors for attachment like intimin and bfpA, along with immunogenic carriers can be potential candidates for vaccine development, given their immune response-generating properties [5]. EPEC vaccines in different stages of development have used multiple platform designs including a whole-cell formalin-killed vaccine, adjuvanted with cholera toxoid, which has shown 100% survival in animal studies [61]. Live attenuated candidate using Citrobacter rodentium strain has elicited effective immune responses in mice following oral administration [62]. Recombinant antigen-based vaccines with mycobacterium species expressing bfpA have produced antibodies along with measurable cytokine response involving TNF-α and INF-γ [5]. The hurdles faced are due to different variants of certain immunogenic proteins like EspB; however, a combination of all variants in a single vaccine has been suggested to test for achieving a successful immune response [4].

Implications for therapeutic strategies

A thorough analysis of the pathogenic effects of E. coli pathotypes will contribute to the production of new therapeutic agents, enhance the production of potent vaccines and ultimately reduce the evolution of the various pathotypes [2]. The scientific and clinical communities have made considerable advancements in comprehending the ecology, pathology, microbiology, and host interactions of *E. coli*. These advancements are essential for developing novel vaccines and therapies to address the severe side effects and challenges of diarrheal diseases caused by *E. coli* [63]. Whole-genome sequencing has provided a wealth of valuable information regarding the genome of pathogenic *E. coli*. This advancement will significantly contribute to managing diseases, epidemiological studies, outbreak investigations, diagnostic processes, classification, and monitoring of pathogen dissemination [14].

Future perspectives and research gaps

There is a growing need for new technologies capable of quickly identifying, characterizing, and monitoring pathogenic *E. coli*. The development of rapid pathogen identification methods is expected to enable clinicians to make faster diagnoses, facilitating timely and appropriate management of patient illness. High-throughput sequencing provides a wealth of data that can address clinical needs, such as identifying potential antibiotic resistance and pathogen-specific virulence factors, supporting epidemiological studies and tracking the spread of pathogens, as extensively discussed in previous reviews [63].

A notable example of the potential and utility of rapid sequencing was demonstrated during the German outbreak of STEC O104:H4. This was further exemplified by assembling a draft genome of STEC O104:H4 using high-throughput sequencing in a retrospective study of stool samples. As demonstrated by previous studies, genome sequence analysis can provide high-resolution typing of epidemic strains, and whole-genome sequencing offers similar detailed insights for typing and outbreak investigations. However, the utility of this data is limited by the need for reliable and meaningful bioinformatics. While a draft genome of a specific pathogen can be generated and sequenced within a couple of days, the nucleic acid data requires thorough quality verification [63].

Additionally, numerous hypothetical genes and genes with unknown functions hinder our understanding of these diseases. Additional genes will likely be identified as more genomic datasets are generated, contributing to the pangenome. The increasing volume of data obtained from whole-genome sequences will enable a deeper understanding of the interactions between viruses and their hosts, their evolution, and the subsequent effects on human health [63]. Although recent progress has been made in understanding the genetic background and pathogenicity of various DEC pathotypes, there remains a need to identify several unique genes encoding unknown activities to comprehend further how these pathogens interact with their hosts [14].

Limitations 

In this study, we have detected certain limitations that should be considered before evaluating its conclusions. Firstly, a systematic review methodology and meta-analysis was not conducted, which could leave out articles with relevant information. Secondly, there could be heterogeneity in the data due to variations in the experimental models and the procedures to identify EPEC which further limited the ability to quantitatively compare results, and no formal evaluation of risk of bias was carried out, as it is a narrative review article. Also, it is possible that more precise conclusions related to each EPEC virulence factor could be made with the presence of quantitative data to allow a more general and specific extrapolation. On the other hand, we know that epidemiological factors differ according to geographical conditions, which could limit the generalization of the findings. Finally, it is considered that in the future, more studies should be developed to delve deeper into the relationship between EPEC and the intestinal microbiota and its impact on the host's immune response. Investigating these interactions could reveal critical mechanisms of pathogenesis, such as how EPEC disrupts gut homeostasis or evades immune defenses and inform the development of novel therapeutic or preventive strategies. Furthermore, future research should aim to standardize methodologies, incorporate large and more diverse datasets, and employ advanced molecular techniques to enhance the reliability, reproducibility, and applicability of findings across different contexts.

## Conclusions

This extensive review can be concluded by highlighting the intricate connection in the EPEC characteristics, epidemiology, host-organism interaction, pathogenesis, virulence factors, and vaccination while comparing its types and how it differs from other enteric pathogens. The diversity of pathogenicity islands and the non-conventional nature of virulence factors make studying them arduous and affect vaccine efficacy. However, advancements in diagnostic guidelines such as molecular techniques and genome sequencing have helped classify and identify diarrheagenic *E. coli*. Substantial studies have been done on intimin, bfpA, and pEAF in the pathogenesis of EPEC infections. Using intimin and bfpA in vaccines can potentially reduce the global burden of EPEC-associated diarrhea; however, no licensed vaccine exists to prevent EPEC infections. Nonetheless, variants are still emerging globally. More studies are needed to study the interaction of EPEC with the intestinal microbiome and immune evasion strategies. In addition, more attempts are needed to enhance immunization coverage by investigating alternate vaccination methods such as a combination of various immunogenic proteins such as EspB in a single vaccine to achieve a successful immune response.

## References

[1] Zhang Y, Tan P, Zhao Y, Ma X (2022). Enterotoxigenic Escherichia coli: intestinal pathogenesis mechanisms and colonization resistance by gut microbiota. Gut Microbes.

[2] Pakbin B, Brück WM, Rossen JW (2021). Virulence factors of enteric pathogenic Escherichia coli: a review. Int J Mol Sci.

[3] Keller Keller, M. and T. Dorr (2025). E. coli is one of the most widely studied organisms - and that may be a problem for both science and medicine. https://theconversation.com/e-coli-is-one-of-the-most-widely-studied-organisms-and-that-may-be-a-problem-for-both-science-and-medicine-206045.

[4] Mare AD, Ciurea CN, Man A (2021). Enteropathogenic Escherichia coli—a summary of the literature. Gastroenterology Insights.

[5] Pokharel P, Dhakal S, Dozois CM (2023). The diversity of Escherichia coli pathotypes and vaccination strategies against this versatile bacterial pathogen. Microorganisms.

[6] Alhadlaq MA, Aljurayyad OI, Almansour A (2024). Overview of pathogenic Escherichia coli, with a focus on Shiga toxin-producing serotypes, global outbreaks (1982-2024) and food safety criteria. Gut Pathog.

[7] Strzelecki P, Karczewska M, Szalewska-Pałasz A, Nowicki D (2025). Phytochemicals controlling enterohemorrhagic Escherichia coli (EHEC) virulence-current knowledge of their mechanisms of action. Int J Mol Sci.

[8] Lee JB, Kim SK, Yoon JW (2022). Pathophysiology of enteropathogenic Escherichia coli during a host infection. J Vet Sci.

[9] Gaytán MO, Martínez-Santos VI, Soto E, González-Pedrajo B (2016). Type three secretion system in attaching and effacing pathogens. Front Cell Infect Microbiol.

[10] Kaur P, Dudeja PK (2023). Pathophysiology of enteropathogenic Escherichia coli-induced diarrhea. Newborn (Clarksville).

[11] Kolodziejek AM, Minnich SA, Hovde CJ (2022). Escherichia coli 0157:H7 virulence factors and the ruminant reservoir. Curr Opin Infect Dis.

[12] Bunduki GK, Heinz E, Phiri VS, Noah P, Feasey N, Musaya J (2021). Virulence factors and antimicrobial resistance of uropathogenic Escherichia coli (UPEC) isolated from urinary tract infections: a systematic review and meta-analysis. BMC Infect Dis.

[13] García A, Fox JG (2021). A one health perspective for defining and deciphering Escherichia coli pathogenic potential in multiple hosts. Comp Med.

[14] Gomes TA, Elias WP, Scaletsky IC (2016). Diarrheagenic Escherichia coli. Braz J Microbiol.

[15] Little JI, Singh PK, Zhao J, Dunn S, Matz H, Donnenberg MS (2024). Type IV pili of Enterobacteriaceae species. EcoSal Plus.

[16] Pawłowska B, Sobieszczańska BM (2017). Intestinal epithelial barrier: the target for pathogenic Escherichia coli. Adv Clin Exp Med.

[17] Snehaa K, Singh T, Dar SA (2021). Typical and atypical enteropathogenic Escherichia coli in children with acute diarrhoea: changing trend in East Delhi. Biomed J.

[18] Singh T, Das S, Ramachandran VG, Shah D, Saha R, Dar SA, Rai A (2017). Typical & atypical enteropathogenic Escherichia coli in diarrhoea & their role as carrier in children under five. Indian J Med Res.

[19] Slinger R, Lau K, Slinger M, Moldovan I, Chan F (2017). Higher atypical enteropathogenic Escherichia coli (a-EPEC) bacterial loads in children with diarrhea are associated with PCR detection of the EHEC factor for adherence 1/lymphocyte inhibitory factor A (efa1/lifa) gene. Ann Clin Microbiol Antimicrob.

[20] Hu J, Torres AG (2015). Enteropathogenic Escherichia coli: foe or innocent bystander?. Clin Microbiol Infect.

[21] Kralicek SE, Sitaraman LM, Kuprys PV, Harrington AT, Ramakrishna B, Osman M, Hecht GA (2022). Clinical manifestations and stool load of atypical enteropathogenic Escherichia coli infections in United States children and adults. Gastroenterology.

[22] Jesser KJ, Levy K (2020). Updates on defining and detecting diarrheagenic Escherichia coli pathotypes. Curr Opin Infect Dis.

[23] Lee W, Sung S, Ha J (2023). Molecular and genomic analysis of the virulence factors and potential transmission of hybrid enteropathogenic and enterotoxigenic Escherichia coli (EPEC/ETEC) strains isolated in South Korea. Int J Mol Sci.

[24] de Graaf M, Beck R, Caccio SM (2017). Sustained fecal-oral human-to-human transmission following a zoonotic event. Curr Opin Virol.

[25] Johnson TJ, Armstrong JR, Johnston B (2022). Occurrence and potential transmission of extended-spectrum beta-lactamase-producing extraintestinal pathogenic and enteropathogenic Escherichia coli in domestic dog faeces from Minnesota. Zoonoses Public Health.

[26] Platenkamp A, Mellies JL (2018). Environment controls LEE regulation in enteropathogenic Escherichia coli. Front Microbiol.

[27] Sora VM, Meroni G, Martino PA, Soggiu A, Bonizzi L, Zecconi A (2021). Extraintestinal pathogenic Escherichia coli: virulence factors and antibiotic resistance. Pathogens.

[28] Zahavi EE, Lieberman JA, Donnenberg MS (2011). Bundle-forming pilus retraction enhances enteropathogenic Escherichia coli infectivity. Mol Biol Cell.

[29] Sun H, Huang D, Pang Y, Chen J, Kang C, Zhao M, Yang B (2024). Key roles of two-component systems in intestinal signal sensing and virulence regulation in enterohemorrhagic Escherichia coli. FEMS Microbiol Rev.

[30] Sy BM, Tree JJ (2020). Small RNA regulation of virulence in pathogenic Escherichia coli. Front Cell Infect Microbiol.

[31] Chen P, Goldberg MB (2023). Recent insights into type-3 secretion system injectisome structure and mechanism of human enteric pathogens. Curr Opin Microbiol.

[32] Zheng M, Sun S, Zhou J, Liu M (2021). Virulence factors impair epithelial junctions during bacterial infection. J Clin Lab Anal.

[33] Lacey CA, Miao EA (2020). Programmed cell death in the evolutionary race against bacterial virulence factors. Cold Spring Harb Perspect Biol.

[34] Navarro-Garcia F, Serapio-Palacios A, Ugalde-Silva P, Tapia-Pastrana G, Chavez-Dueñas L (2013). Actin cytoskeleton manipulation by effector proteins secreted by diarrheagenic Escherichia coli pathotypes. Biomed Res Int.

[35] de Jong MF, Alto NM (2018). Cooperative immune suppression by Escherichia coli and Shigella effector proteins. Infect Immun.

[36] Lee PC, Rietsch A (2015). Fueling type III secretion. Trends Microbiol.

[37] Murashko ON, Lin-Chao S (2017). Escherichia coli responds to environmental changes using enolasic degradosomes and stabilized DicF sRNA to alter cellular morphology. Proc Natl Acad Sci U S A.

[38] Balasubramanian D, Ragunathan PT, Fei J, Vanderpool CK (2016). A prophage-encoded small RNA controls metabolism and cell division in Escherichia coli. mSystems.

[39] Donnenberg MS, Finlay BB (2013). Combating enteropathogenic Escherichia coli (EPEC) infections: the way forward. Trends Microbiol.

[40] Law RJ, Gur-Arie L, Rosenshine I, Finlay BB (2013). In vitro and in vivo model systems for studying enteropathogenic Escherichia coli infections. Cold Spring Harb Perspect Med.

[41] Desvaux M, Dalmasso G, Beyrouthy R, Barnich N, Delmas J, Bonnet R (2020). Pathogenicity factors of genomic islands in intestinal and extraintestinal Escherichia coli. Front Microbiol.

[42] Miner MV, Rauch I (2024). Why put yourself on a pedestal? The pathogenic role of the A/E pedestal. Infect Immun.

[43] Singh AP, Aijaz S (2015). Enteropathogenic E. coli: breaking the intestinal tight junction barrier. F1000Res.

[44] Yen H, Karino M, Tobe T (2016). Modulation of the inflammasome signaling pathway by enteropathogenic and enterohemorrhagic Escherichia coli. Front Cell Infect Microbiol.

[45] Govindarajan D.K, Viswalingam N, Meganathan Y (2020). Adherence patterns of Escherichia coli in the intestine and its role in pathogenesis. Med Microecol.

[46] Ray A, Moore TF, Pandit R, Burke AD, Borsch DM (2023). An overview of selected bacterial infections in cancer, their virulence factors, and some aspects of infection management. Biology (Basel).

[47] Figaj D, Ambroziak P, Rzepka I, Skórko-Glonek J (2022). SurA-like and Skp-like proteins as important virulence determinants of the gram negative bacterial pathogens. Int J Mol Sci.

[48] Sanchez-Garrido J, Slater SL, Clements A, Shenoy AR, Frankel G (2020). Vying for the control of inflammasomes: the cytosolic frontier of enteric bacterial pathogen-host interactions. Cell Microbiol.

[49] Pinaud L, Sansonetti PJ, Phalipon A (2018). Host cell targeting by enteropathogenic bacteria T3SS effectors. Trends Microbiol.

[50] Rao S, Schieber AM, O'Connor CP, Leblanc M, Michel D, Ayres JS (2017). Pathogen-mediated inhibition of anorexia promotes host survival and transmission. Cell.

[51] Pallett MA, Crepin VF, Serafini N (2017). Bacterial virulence factor inhibits caspase-4/11 activation in intestinal epithelial cells. Mucosal Immunol.

[52] Kobayashi T, Ogawa M, Sanada T (2013). The Shigella OspC3 effector inhibits caspase-4, antagonizes inflammatory cell death, and promotes epithelial infection. Cell Host Microbe.

[53] Chung LK, Park YH, Zheng Y (2016). The Yersinia virulence factor YopM hijacks host kinases to inhibit type III effector-triggered activation of the pyrin inflammasome. Cell Host Microbe.

[54] Clark SE, Schmidt RL, McDermott DS, Lenz LL (2018). A Batf3/Nlrp3/IL-18 axis promotes natural killer cell IL-10 production during listeria monocytogenes infection. Cell Rep.

[55] Semper RP, Vieth M, Gerhard M, Mejías-Luque R (2019). Helicobacter pylori exploits the NLRC4 inflammasome to dampen host defenses. J Immunol.

[56] Nara JM, Cianciarullo AM, Culler HF (2010). Differentiation of typical and atypical enteropathogenic Escherichia coli using colony immunoblot for detection of bundle-forming pilus expression. J Appl Microbiol.

[57] Ochoa TJ, Contreras CA (2011). Enteropathogenic escherichia coli infection in children. Curr Opin Infect Dis.

[58] Zollner-Schwetz I, Krause R (2015). Therapy of acute gastroenteritis: role of antibiotics. Clin Microbiol Infect.

[59] Jafari E, Mostaan S, Bouzari S (2020). Characterization of antimicrobial susceptibility, extended-spectrum β-lactamase genes and phylogenetic groups of enteropathogenic Escherichia coli isolated from patients with diarrhea. Osong Public Health Res Perspect.

[60] Eltai NO, Al Thani AA, Al Hadidi SH, Al Ansari K, Yassine HM (2020). Antibiotic resistance and virulence patterns of pathogenic Escherichia coli strains associated with acute gastroenteritis among children in Qatar. BMC Microbiol.

[61] Gohar A, Abdeltawab NF, Fahmy A, Amin MA (2016). Development of safe, effective and immunogenic vaccine candidate for diarrheagenic Escherichia coli main pathotypes in a mouse model. BMC Res Notes.

[62] Wang S, Xia X, Liu Y, Wan F (2022). Oral administration with live attenuated Citrobacter rodentium protects immunocompromised mice from lethal infection. Infect Immun.

[63] Croxen MA, Law RJ, Scholz R, Keeney KM, Wlodarska M, Finlay BB (2013). Recent advances in understanding enteric pathogenic Escherichia coli. Clin Microbiol Rev.

